# Prevalence and Determinants of Undernutrition among 6- to 59-Months-Old Children in Lowland and Highland Areas in Kilosa District, Tanzania: A Cross-Sectional Study

**DOI:** 10.1155/2021/6627557

**Published:** 2021-04-11

**Authors:** Jackline D. Mrema, Ester Elisaria, Akwilina W. Mwanri, Cornelio M. Nyaruhucha

**Affiliations:** ^1^Ifakara Health Institute, Department of Health System, Impact Evaluation and Policy, P.O. Box 78373, Dar Es Salaam, Tanzania; ^2^Department of Food Technology, Nutrition and Consumer Sciences, Sokoine University of Agriculture, P.O. Box 3006, Morogoro, Tanzania

## Abstract

**Background:**

Undernutrition is the most dominant form of malnutrition among children in developing countries. Studies conducted in Tanzania have reported high levels of undernutrition among children below five years of age. However, there is limited information on differences in stunting prevalence across agroecological zones. This study aimed to determine the prevalence of undernutrition and its determinants in the lowland and highland areas in Kilosa District, Tanzania.

**Methods:**

A cross-sectional study was conducted in a sample of 200 randomly selected households from the lowland and 141 from the highland areas of Kilosa District in Morogoro Region, Tanzania. Sociodemographic, feeding practices, hygiene, and sanitation data were collected using a structured questionnaire. Weight and height of children were measured using a standard procedure, and age was calculated from the birth date obtained from the child growth card. Anthropometric data were analyzed by using Emergency Nutrition Assessment (ENA) software. The logistic regression model was used to explore the determinants of undernutrition.

**Results:**

Prevalence of stunting, underweight, and wasting was 41.0%, 11.5%, and 2.5% in lowland and 64.5%, 22.0%, and 1.4% in highland areas, respectively. The prevalence of stunting and underweight was higher in the highland compared to the lowland areas (*p* < 0.001). Significant determinants of underweight were areas of residence (AOR 4.21, 95% CI: 1.62–10.9), age of the children (AOR 5.85, 95% CI: 1.81–18.97), and child birth weight (AOR, 4.98 95% CI: 1.65–15.05), while determinants of stunting were the area of residence (AOR, 2.77 95% CI: 1.43–5.36), maternal age (AOR, 0.33 95% CI: 0.14–0.79), sex of a child (AOR, 1.89 95% CI: 1.03–3.50), and child birth weight (AOR, 3.29 95% CI: 1.21–8.97).

**Conclusion:**

The prevalence of undernutrition, especially stunting and underweight, was high in the study areas. Determinants of stunting differed between highlands and lowland areas, highlighting the needs of having properly integrated interventions based on the geographical location.

## 1. Introduction

Malnutrition is the leading cause of morbidity in children under five years of age, where globally 150.8 and 50.5 million children were stunted and wasted, respectively, in 2017 [1]. In Africa, the prevalence of stunted children decreases from 38.3% in 2012 to 30.3% in 2017, although the actual number of stunted children has risen due to population growth [1]. Studies conducted in Tanzania indicated malnutrition among children under five years is in a declining trend but still high as per WHO standards [2–4]. As of 2015, 34% of children below five years of age were stunted at the country level, which is 8% points lower than what was reported in 2010 [4]. The prevalence of underweight and wasting was 14% and 5%, respectively. The country experiences a large variation of the burden of malnutrition across regions with high food-producing areas being the most affected [4]. The prevalence of undernutrition for children below five years of age in the Morogoro Region is still high, 33.4% are stunted, 11.5% are underweight, and 6% are wasted [4].

A study conducted in Iran reported household income as a contributing factor for undernutrition, whereby the rate of undernutrition was inversely associated with income [5]. Another study conducted in Serbia by Janevic et al. [6] reported factors such as inadequate dietary intake and lack of essential health services, as well as poor hygiene and sanitation. In Iran, Ethiopia, Nigeria, and Kenya, the risks of becoming undernourished were the sex of the child, maternal education, child age, and household size [5, 7–9]. Besides, hygiene and sanitation play a major role in the nutritional status of the child and lack of safe water affects negatively hygiene and sanitation, as well as increases the risk to acquire water-borne diseases [5]. In studies conducted in Nepal and Kenya, factors observed were birth order, child spacing, early-recommended complementary foods, insufficient energy and micronutrient intake, and low birth weight [10, 11].

Few studies conducted in urban and rural areas of Tanzania reported sociodemographic, infant and young child feeding practices, and maternal characteristics, as well as water hygiene and sanitation, as factors for undernutrition [3, 12, 13]. Other factors reported were size of the farm, sex, and age of the child, inappropriate feeding practices such as delaying the onset of breastfeeding and prelacteal feeding, duration of breastfeeding, household family size, use of iodized salt, distance to the water source, inadequate dietary intake before conception or during pregnancy, and the mother's body mass index (BMI), age, and literacy. Other determinants include diarrhea, water supply, and improved latrine [3, 12, 13].

Nevertheless, there is limited information on the nutritional status of children by topographical differences (i.e., children residing in the lowland and highland areas). Most of the studies conducted in other countries which compared the nutritional status of the children in lowland and highland areas came up with contradicting results. For example, studies in Ecuador and Yemen showed that children in the highlands were more malnourished than those in the lowlands, while other authors reported that children in the lowlands were more malnourished compared to their peers in the highlands [14, 15]. This study aimed at determining the prevalence and determinants of undernutrition in children residing in lowland and highland areas of the Kilosa District, Morogoro Region.

## 2. Materials and Methods

### 2.1. Study Design and Settings

A household cross-sectional survey was conducted from December 2016 to January 2017 in a random sample of five villages of Kilosa District, namely, Chanzuru, Peapea, and Batini from lowland and Unone and Mfuluni from highland areas. Kilosa District is located in East-Central Tanzania, about 148 km from Morogoro town. It extends between latitude 5°55′ and 7°53′ south and longitude 36°30′ and 37°30′ east [16]. The topography of the district contains highland (865 meters above sea level) and lowland (437 meters above sea level) areas with agriculture being the most economic activity.

### 2.2. Respondents and Eligibility Criteria

All mothers or caregivers of children aged 6–59 months and their children in the selected villages were eligible. Women with the youngest child were selected for the interview in households with multiple women having children aged 6–59 months to reduce recall bias. All children with malformations, sick, and/or those whose mothers refused to participate were excluded from the study. The purpose of the study was explained to all respondents, and written informed consent was obtained before the interview.

### 2.3. Sample Size and Sampling

The study sample size was determined using Fisher's Formula [17].

(1) $$\begin{array}{c} n=z^{2}\frac{pq}{d^{2}}, \end{array}$$

where *n* = the desired sample and *z* = the standard normal deviate (which is 1.96 corresponding to 95% CI). *p* = proportion of prevalence of stunting in Morogoro Region (prevalence of stunting in Morogoro was 33.4% = 0.334). *q*=1 − *p*(1−0.334=0.666). *d* = degree of accuracy desired (0.05).

Calculating the value of *n*, *n* = 1.96^2^ ∗ 0.334 ∗ 0.666/0.05^2^, *n*=341.

Therefore, a total of 341 households were required. A two-stage stratified sampling strategy was employed by selecting villages at the first stage and households at the second stage proportional to the population size of the strata (highland or lowland). Lowland areas had large population size in comparison to highland areas which resulted in more households being selected in lowland areas. A list of households with children aged 6–59 months prepared by the village leader residing in the selected villages was used as a sampling frame for second-stage sampling. Approximately 68 households were sampled from each of the five villages selected.

### 2.4. Data Collection Methods and Instruments

A structured questionnaire was used to interview mothers/caregivers of children aged 6–59 months and document the values of their anthropometric measurements. The altitude was captured using GPS machines. The questionnaire captured sociodemographic characteristics, child feeding practices (breastfeeding and complementary feeding), environmental sanitation and personal hygiene, and child weight and height measurements. Weight was measured using an electronic SECA weighing scale (seca GmbH & Co. KG, Hamburg, Germany) to the nearest 0.1 kg. The child clothes were reduced as much as possible and shoes were removed during measurement. The scale was placed on a flat surface area, and children aged 2 years and above who were able to stand were weighed while standing on a scale. Children who could not stand alone had their mother stood on the scale first and the reading reset; then, the mother handled the child to have the child reading recorded automatically on the scale. Height was measured by using a stadiometer (Shorr Board ICA 420) in the supine position or recumbent position of the child below two years. A child with more than two years was measured while standing on a stadiometer without shoes, and the measurement was recorded to the nearest 0.1 cm. The nutritional status was classified using the WHO classification [18]. Adherence to the measuring techniques and recording procedures was observed to reduce measurement errors.

### 2.5. Data Processing and Analysis

Anthropometric data were processed using ENA for SMART (2008) software and analysed using Statistical Package for the Social Sciences (SPSS) software version 20. Frequencies, percentages, and means were used for descriptive analysis. Weight and height measurements were converted into three summary indices of nutritional status, weight-for-age (underweight), height-for-age (stunting), and weight-for-height (wasting), according to the 2006 WHO criterion-based *Z* scores [19]. Wasting was defined as a weight-for-height *Z* score of less than −2 of the WHO child growth standards median, underweight as a weight-for-age *Z* score of less than −2 of the WHO child growth standards median, and stunting as a height-for-age *Z* score of less than −2 of the WHO child growth standards median. A two-sample *t*-test was used to compare these nutrition indicators between lowland and highland areas. Logistic regression analysis was performed to assess the potential determinants of undernutrition. Predictors of child nutrition status (stunting and underweight) as outcome variables were explored at the general population and in each location (lowland and highland) separately. Due to the small sample size, stratified multivariate logistic regression models were not fitted as they will give bias results with low reliability (wide confidence intervals). Breastfeeding behaviours (initiation and exclusive breastfeeding), dietary diversity, child age, sex and birth weight, maternal age, and marital status and sex of the head of the household were considered in building the overall model for both outcome variables.

## 3. Results

### 3.1. Demographic Characteristics of Study Participants

A total of 341 respondents, 200 from lowland and 141 from highland areas of Kilosa District, were interviewed. A majority of the mothers (82.1%) were married, and 74% had primary school as the highest level of education. More than 80% of the households were male-headed. The main source of income in both areas was agriculture followed by casual labor. Mother/caregiver ages ranged from 16 to 68 years with a mean of 28.4 years. Nearly half (46.9%) of them were aged between 25–34 years. The age of the children recruited ranges from 6–59 months with a mean age of 31.1 months (Table 1).

### 3.2. Young Child Feeding Practices

Table 2 shows the infant and young child feeding practices. Almost all children were ever breastfed; 74% were exclusively breastfed for six months in lowlands while 65.2% in the highland areas. Overall, more than half of the children (57.2%) were breastfed within one hour of birth. Over two-thirds (66.7%) of the children were breastfed within one hour of birth in the lowland compared to 48% of the children in the highland areas. About 30% of mothers introduced complementary foods to their children before the age of six months, with some starting as early as during the first month of life. Eight out of every ten children (80.6%) consumed less than four food groups, and only 19.4% met the minimum dietary diversity of four or more food groups. Children in the lowland area ate more diversified food (23%) than those in the highland area (14.2%).

### 3.3. Children's Nutritional Status

The nutritional status of children in lowland and highland areas is shown in Table 3. The overall prevalence of underweight in the Kilosa District was 15.8, whereby the prevalence in highland was about twice (22%) of that in the lowland area (11.5%). Half of the children (50.7%) in the study district were stunted, and severely stunted children in highland (29%) areas doubled that of lowland areas (13.5%). The prevalence of wasting was slightly higher in lowlands compared to highland areas (2.5 and 1.4%, respectively). There was a significant difference in weight-for-age and height-for-age indices between the children in lowland and highland (*p* < 0.001).

### 3.4. Determinants of Underweight in Children

Area of residence, age, and birth weight of the child were significant determinants for underweight (Table 4). Children who lived in the highland areas were two times more likely to be underweight (OR 2.17, 95% CI: 1.20–3.91) compared to children in the lowland areas. Children aged between 36–59 months were two times more likely to be underweight (OR 2.14, 95% CI: 1.04–4.39) compared to younger children (6–23 months). Children with low birth weight (<2.5 kg) were three times more likely to be underweight (OR 2.95, 95% CI: 1.37–6.34) compared to those with normal birth weight (≥2.5 kg). Adjusting for all factors in Table 4 increases the risk for underweight in the highland area from OR 2.17, 95% CI: 1.20–3.91 to AOR 4.21, 95% CI: 1.62–10.9. Furthermore, children's age and birth weights were significantly associated with underweight with AOR 5.85, 95% CI: 1.81–18.97 and AOR 4.98, 95% CI: 1.65–15.05, respectively.

### 3.5. Determinants of Stunting

Area of residence, maternal marital status, and sex of the household head were significant determinants for stunting (Table 5). Children living in the highland areas were almost three times as likely to be stunted (OR 2.62, 95% CI: 1.68–4.09) compared to those in the lowland areas. Children living with a single parent (mother only) had an increased risk of stunting (OR 2.11, 95% CI: 1.18–3.75). After adjusting for maternal age, marital status, sex, and childbirth weight, the area of residence was still a significant determinant for stunting (AOR, 2.77 95% CI: 1.43–5.36).

### 3.6. Determinants of Stunting in Lowland and Highland Areas

Table 6 presents factors associated with stunting in the highland and lowland areas. Significant factors for stunting in the lowland area were marital status, sex of household head, child age, and maternal age. Children living with mothers only (OR 2.61 (1.21–5.60)) and female-headed households (OR 2.27 (1.04–4.95)) were more likely to be stunted than those living with married mothers and in male-headed households. Moreover, young children were more stunted compared to older (aged 36–59 months) children. Furthermore, children whose mothers aged between 25–34 years had a reduced risk of being stunted (OR 0.37 (0.19–0.74)) compared to the children whose mothers aged between 14–24 years.

In the highland area, a significant determinant for stunting was only maternal age. Mothers aged between 25–34 years had a reduced risk of having a stunted child (OR 0.35, 95% CI: 0.16–0.76) compared to the children whose mothers aged between 14–24 years.

## 4. Discussion

This study assessed the prevalence and determinants of undernutrition among 6–59-month-old children in lowland and highland areas of Kilosa District in Morogoro. The overall prevalence of underweight was 15.8%, similar to the national average [4]. Location wise, highland areas have a high prevalence of underweight children compared to lowland areas. Being one of the rural districts, the high prevalence observed could be explained by food insecurity, poverty, or a short-term inadequate intake, which might have been contributed by the timing of data collection. This study was conducted during a lean season where most of the households had finished stock of their food. It has been reported elsewhere that child nutritional status varies by season, with a relatively high prevalence of underweight in the lean season compared to the harvest season [20]. Studies conducted in other rural districts of Tanzania showed a high prevalence of underweight [3, 21]. According to WHO [22] category, the study subjects were in medium rates (10–19%) of underweight in the lowland area and high rates (20–29%) in the highland area. The results concur with a study conducted in Ecuador which found that children in the highland areas were more underweight than those in the lowland areas [15].

The overall prevalence of stunting was 50.7%, higher than the WHO threshold of 40% [21, 23] and compared to the national average (34%) and that of Morogoro Region (33.4%) [4]. Higher prevalence was found in the highland area, similar to the results reported by UNICEF in the Hijja mountains in Yemen [14]. The high prevalence observed in these settings could be due to poor child feeding practices, delayed breastfeeding initiation, early complementation, and inadequate feeding. These factors showed an increased risk of stunting but were not statistically significant in the crude analysis due to the small sample size. About 40% of the children were initiated on breastfeeding after one hour of birth while 30% were not exclusively breastfed as recommended by the WHO. However, this might be exaggerated with maternal recall bias as these questions were asked retrospectively. Delaying initiation of breastfeeding deprives the infant nutritional benefit of the colostrum and may impede nutritional status. Inappropriate breastfeeding and delayed breastfeeding initiation have been previously reported in Tanzania [3]. Inadequate feeding practices could also be attributed to household poverty and lack of feeding knowledge. Most children in Kilosa (80.6%) were fed a less diversified diet. Similar findings were reported by Kulwa et al. in a study conducted in rural central Tanzania [23]. The stunting rates, especially in highland areas, call for an integrated response to reduce the prevalence and the resultant long-term effects of stunting.

Area of residence was significantly associated with underweight. Children living in the highlands were two times more likely to be underweight compared to those in the lowland areas. People living in the highland area are likely to have poor access to health services, poor infrastructure, and long distances to access a health facility, which might hinder the use of ANC and postnatal care. Poor use of ANC and postnatal-care services may have contributed to the observed high prevalence of stunting since child vaccination strengthens children's immunity and reduces the risk of infection [24]. Highland areas are known to have poor-quality soil [25] which could lead to low agricultural production and poor quality of products, eventually affecting the dietary intake of residents. A child's age was also a significant determinant of underweight where a high proportion of older children (35–59 months) were underweight compared to younger children (6–23 months). Small feeding frequency was likely to be one of the possible explanations as the majority of children were reported to be given two to three meals per day instead of three to four meals plus two healthy snacks as recommended by WHO [26]. In addition, the only source of nutrients for older children was complementary foods, while the younger children benefited from both breastmilk and complementary food. These factors might have compromised the child nutritional status as at this age, children undergo rapid growth which creates a high demand for energy and other nutrients [27, 28]. Birth weight was also a significant determinant for underweight, with children born with low birth weight (<2.5 kg) being three times more likely to be underweight compared to the normal birth babies (≥2.5 kg). Children with low birth weight are often disadvantaged in terms of physical growth and are also more vulnerable to infections compared to children born with normal birth weight [29]. Other researchers also found an association between low birth weight and undernutrition later in life [30, 31]. Another study conducted in Kilosa District reported an association of malaria with underweight [32]; hence, the high prevalence of underweight could be a result of the endemic malaria parasite in the study area.

Children living in highland areas were twice more likely to be stunted compared to those living in the lowland areas. Stunting as a result of poor infant feeding might manifest later in life since it is a chronic condition. The recorded stunting level might be due to poor dietary diversity whereas 86% of children in the highlands consumed less than four food groups compared to 77% in the lowlands. The World Health Organization (WHO) recommends that children should be fed at least four food groups per meal [26]. Furthermore, it might be due to food insecurity as a result of poor soil quality [25]. Studies carried out in Ecuador and Guatemala also reported that area of residence was a significant determinant of stunting [15]. Another determinant was marital status where children who live with a single parent (mother only) were twice more likely to be stunted compared to those who lived with both parents (married mothers). Moreover, children from female-headed households were more likely to be stunted. This could be due to the high burden of responsibility posed to mothers, including reproduction, production, household chores, and child care. Similar findings were reported after the reanalysis of the Tanzania Demographic Health Survey (DHS) from 1991–2016 [33]. The risk for stunting was reduced in children living with mothers aged 25 years and above compared to mothers aged less than 25 years. Teenage mothers have less power to provide a child with an adequate diet, safe water, and sanitary services due to inadequate financial resources [34]. They also have a high demand for nutrients to support the growth and development of foetus and, hence, more likely to give birth to a low birth weight baby. As a result of these challenges, quality of care, child feeding, and nurturing may be compromised, leading to high rates of childhood stunting compared to children from adult mothers [34]. Birth weight was another significant determinant of stunting. Children with low birth weight were three times more likely to be stunted compared to those with normal birth weight. The link between low birth weight and child malnutrition could be attributed to the increased vulnerability of children with low birth weight to infections. Several studies have reported high rates of undernutrition in children with low birth weight [35–37]. Another significant determinant of stunting was the sex of the children. Boys were more likely to be stunted compared to girls. This could be due to cultural beliefs that attribute higher appetite and low satiety to boys compared to girls. It is believed that boys are often not fully satisfied with breastmilk and need to be introduced to supplementary foods at an early age [38], which increases the risk of diarrhea and, hence, stunting. The gender differences could also be due to behavioural patterns employed by girls such as spending more time with their mothers in the kitchen compared to boys who are hyperactive and spend most of their time playing outside home where they can skip several meals. This finding was similar to that of Babatunde et al. and Chirande et al. [12, 39] who reported boys being more stunted compared to girls. On exploring factors related to stunting in the two study settings, only maternal age showed an association with stunting in the highland areas, while in the lowland areas, four key determinants were related to stunting (maternal marital status and sex of head of the household head, as well as child and maternal age).

## 5. Conclusions

Stunting and underweight were higher in children living in highland areas compared to children in lowland areas. Maternal age was the only significant determinant of stunting in both lowland and highland areas. The Ministry of Health, Community Development, Gender, Elderly, and Children needs to establish context-specific policies and programs targeting child malnutrition because of the diverse nature of the factors that impact child nutrition in their first two years of life. Government and nongovernment organizations should also establish special programs and campaigns to combat teenage pregnancies while considering differences in access to health services between lowland and highland areas.

This study has some limitations. Firstly, a large area of Kilosa District is lowland due to its topographical setup, resulting in a bigger sample being taken from lowland areas. This may have affected the comparison of indicators between lowland and highland areas. Secondly, the recall bias might have happened, especially when capturing breastfeeding information retrospectively when interviewing mothers and caregivers. Furthermore, it is important to consider differences in geographical location, access to health services, and economic status when trying to generalize the results in other settings.

## Figures and Tables

**Table 1:** Demographic characteristics of the studied population in Kilosa District (*N* = 341).

Demographic information	Lowland (*N* = 200)		Highland (*N* = 141)		Total (*N* = 341)	
	*n*	%	*n*	%	*N*	%
*Marital status*						
Single	31	15.5	25	17.7	56	16.4
Married	167	83.5	113	80.1	280	82.1
Divorced	2	1	3	2	5	1.5

*Maternal education level*						
Informal education	36	18	25	17.7	61	17.9
Primary	147	73.5	107	75.9	254	74.5
Secondary/university	17	8.5	9	6.4	26	7.6

*Occupation of the mother*						
Farmer	185	92.5	139	98.6	324	95
Employed	5	2.5	0	0	5	1.5
Petty business	10	5.0	2	1.4	12	3.5

*Sex of children*						
Male	106	53	68	48.2	174	51
Female	94	47	73	51.8	167	49

*Children ages*						
6–23 months	63	31.5	51	36.2	114	33.4
24–35 months	58	29	44	31.2	102	29.9
36–59 months	79	39.5	46	32.6	125	36.7

*N* = total number of respondents, *n* = total number of respondents in each category, % is presented as a column.

**Table 2:** Young children feeding practices in the lowland and highland areas (*n* = 341).

Variable	Lowland (*N* = 200)		Highland (*N* = 141)		Total (*N* = 341)	
	*n*	%	*n*	%	*n*	%
*Breastfeeding*						
Never breastfed	2	1	0	0	2	0.6
Ever breastfed	198	99	141	100	339	99.4

*Timing of breastfeeding initiation*						
Within 1 hour	132	66.7	63	47.7	199	57.2
1–6 hours	64	32.3	69	48.9	134	39
More than 12 hours	2	1	2	1.4	4	1.2
I don't know	2	1	7	5	9	2.6

*Frequency of breastfeeding/day*						
Anytime	195	97.5	138	97.9	333	97.7
Twice	0	0	1	0.7	1	0.3
Three times	1	0.5	0	0	1	0.3
Four times	4	2	2	1.4	6	1.7

*Exclusive breastfeeding* (6 *months*)						
No	52	26	49	34.8	101	29.6
Yes	148	74	92	65.2	240	70.4

*Time when complementary food was started*						
<6 months	52	26	49	34.8	101	29.6
At 6 months	138	69	87	61.7	225	66
>6 months	10	5	5	3.5	15	4.4

*No. of meals per day*						
One	6	3	2	1.4	8	2.4
Two meals	25	12.5	23	16.3	48	14.1
Three meals	134	67	109	77.3	243	71.2
Four or more meals	35	17.5	7	5	42	12.3

*Dietary diversity scores*						
<4 food groups	154	77	121	85.8	275	80.6
≥4 groups and above	46	23	20	14.2	66	19.4

*N* = total number of respondents, *n* = number of respondents in each category, % is presented as a column.

**Table 3:** Child nutritional status in lowland and highland areas.

	Location				Overall		*p* value^*∗*^
	Lowland		Highland		Prevalence *n* (%)		
	*n*	%	*n*	%	*n*	%	
*Weight-for-Age* (*WAZ*)							
Severe underweight	3	1.5	6	4.3	9	2.6	<0.001
Moderate underweight	20	10	25	17.7	45	13.2	
Overall underweight	23	11.5	31	22	54	15.8	
Normal	177	88.5	110	78	287	84.2	

*Height-for-Age* (*HAZ*)							
Severe stunting	27	13.5	41	29	68	19.9	<0.001
Moderate stunting	55	27.5	50	35.5	105	30.8	
Overall stunting	82	41	92	64.5	173	50.7	
Normal	118	59	50	35.5	168	49.3	

*Weight-for-Height* (*WHZ*)							
Severe wasting	3	1.5	1	0.7	4	1.2	0.91
Moderate wasting	2	1	1	0.7	3	0.9	
Overall wasting	5	2.5	2	1.4	7	2.1	
Normal	195	97.5	139	98.6	334	97.9	

^*∗*^The continuous variables calculated from HAZ, WHZ, and WAZ, *n* = number of children with outcome of interest, % is presented as a column.

**Table 4:** Determinants of underweight in children: crude and adjusted OR.

Variable	Underweight			Crude OR (CI)	Adjusted OR (CI)
	*N*	*n*	%		
*Location*					
Lowland	200	23	11.5	1	—
Highland	141	31	22	2.17 (1.20–3.91)^∗∗^	4.21 (1.62–10.91)^∗∗^

*Marital status*					
Single	61	9	14.8	1	—
Married	280	45	16.1	1.11 (0.51–2.40)	2.48 (0.60–10.21)

*Sex of the child*					
Male	174	30	17.2	1.24 (0.69–2.23)	1.56 (0.67–3.65)
Female	167	24	14.4	1	—

*Child age*					
6–23 months	114	13	11.4	1	—
25–35 months	102	14	13.7	1.24 (0.55–2.77)	1.99 (0.54–7.41)
36–59 months	125	27	21.6	2.14 (1.04–4.39)^*∗*^	5.85 (1.81–18.97)^*∗*^

*Birth weight*					
<2.5	38	12	31.6	2.95 (1.37–6.34) ^∗∗^	4.98 (1.65–15.05) ^∗∗^
≥2.5	281	38	13.5	1	—

^*∗*^Significant at *p* < 0.05; ^∗∗^significant at *p* < 0.01. *N* = total number of respondents, *n* = number of underweight children, % is presented as a column.

**Table 5:** Determinants of stunting in children: crude and adjusted OR (*N* = 341).

Variable	Stunting			Crude OR (CI)	Adjusted OR (CI)
	*N*	*n*	%		
*Location*					
Lowland	200	82	41.0	1	1
Highland	141	91	64.5	2.62 (1.68–4.08)^∗∗∗^	2.77 (1.43–5.36)^∗∗^

*Marital status*					
Single	61	40	65.6	2.11 (1.18–3.75)^∗∗^	1.84 (0.79–4.28)
Married	280	133	47.5	1	1

*Age of the mother*					
14–24	113	74	65.5	1.69 (0.91–3.12)	1.58 (0.49–5.09)
25–34	160	63	39.4	0.58 (0.33–1.02)	0.33 (0.14–0.79)^*∗*^
35–70	68	36	52.9	1	1

*Sex of the HH*					
Male	284	136	47.9	1	
Female	57	37	64.9	2.01 (1.11–3.64)^*∗*^	

*Sex of the child*					
Male	174	97	55.7	1.51 (0.98–2.31)	1.89 (1.03–3.50)^*∗*^
Female	167	76	45.5	1	1

*Birth weight*					
<2.5	38	21	55.3	1.24 (0.6–2.5)	3.29 (1.21–8.97)^∗∗^
≥2.5	281	140	49.8	1	1

^*∗*^Significant at *p* < 0.05; ^∗∗^significant at *p* < 0.01, ^∗∗∗^significant at *p* < 0.001, *N *= total number of respondents, *n* = number of stunted children, % is presented as a column.

**Table 6:** Determinants of stunting in lowlands and highlands.

Variable	OR at lowlands (CI) *N* = 200	OR at highlands (CI) *N* = 141
*Marital status*		
Single	2.61 (1.21–5.60)^*∗*^	1.48 (0.59–3.60)
Married	1	1

*Age of the mother*		
14–24	1	1
25–34	0.37 (0.19–0.74)^*∗*^	0.35 (0.16–0.76)^*∗*^
35–70	0.62 (0.28–1.36)	0.87 (0.29–2.65)

*Sex of HH*		
Male	1	1
Female	2.27 (1.04–4.95)^*∗*^	1.62 (0.63–4.17)

*Sex of the child*		
Male	1.73 (0.98–3.07)	1.48 (0.74–2.96)
Female	1	1

*Child age*		
6–23	2.08 (1.05–4.15)^*∗*^	0.97 (0.41–2.26)
24–35	2.14 (1.06–4.32)^*∗*^	0.69 (0.29–1.67)
36–59	1	1

^*∗*^Significant at *p* < 0.05, *N* = total number of children.

## Data Availability

The datasets used during the current study are available from the corresponding author on request.

## Abbreviations

AOR: Adjusted odds ratio
CI: Confidence interval
COR: Crude odds ratio
HAZ: Height-for-Age *Z* score
TDHS: Tanzania demographic and health survey
WAZ: Weight-for-Age *Z* score
WHO: World Health Organization
WHZ: Weight-for-Height *Z* score.

## References

[1] Fanzo J., Hawkes C., Udomkesmalee E. (2018). The 2018 global nutrition report: shining a light to spur action on nutrition.

[2] Mamiro P. S., Kolsteren P., Roberfroid D., Tatala S., Opsomer A. S., Van Camp J. H. (2005). Feeding practices and factors contributing to wasting, stunting, and iron-deficiency anaemia among 3–23-month old children in Kilosa district, rural Tanzania. *Journal of Health Population and Nutrition*.

[3] Safari J. G., Masanyiwa Z. S., Lwelamira J. E. (2015). Prevalence and factors associated with child malnutrition in Nzega district, rural Tanzania. *Current Research Journal of Social Sciences*.

[4] Ministry of Health and Social Welfare (2016). *Tanzania Demographic and Health Survey and Malaria Indicator Survey (TDHS-MIS) 2015-16*.

[5] Kavosi E., Hassanzadeh Rostami Z., Nasihatkon A., Moghadami M., Heidari M., Kavosi Z. (2014). Prevalence and determinants of under-nutrition among children under six: a cross-sectional survey in Fars province, Iran. *International Journal of Health Policy and Management*.

[6] Janevic T., Petrovic O., Bjelic I., Kubera A. (2010). Risk factors for childhood malnutrition in Roma settlements in Serbia. *BMC Public Health*.

[7] Badake Q. D., Immaculate M., Mboganie M. A. (2014). Nutritional status of children under five years and associated factors in Mbeere South district, Kenya. *African Crop Science Journal*.

[8] Fentahun W., Wubshet M., Tariku A. (2016). Undernutrition and associated factors among children aged 6–59 months in East Belesa district, northwest Ethiopia: a community based cross-sectional study. *BMC Public Health*.

[9] Senbanjo I. O., Olayiwola I. O., Afolabi W. A. O. (2016). Dietary practices and nutritional status of under-five children in rural and urban communities of Lagos state, Nigeria. *Nigerian Medical Journal*.

[10] Masibo P. K., Makoka D. (2012). Trends and determinants of undernutrition among young Kenyan children: Kenya demographic and health survey; 1993, 1998, 2003 and 2008-2009. *Public Health Nutrution*.

[11] Bhandari T. R., Chhetri M. (2013). Nutritional status of under five year children and factors associated in Kapilvastu district, Nepal. *Journal of Nutritional Health and Food Science*.

[12] Chirande L., Charwe D., Mbwana H. (2015). Determinants of stunting and severe stunting among under-fives in Tanzania: evidence from the 2010 cross-sectional household survey. *BMC Pediatrics*.

[13] Mbwana H. A., Kinabo J., Lambert C., Biesalski H. K. (2017). Factors influencing stunting among children in rural Tanzania: an agro-climatic zone perspective. *Food Security*.

[14] UNICEF (2012). *Nutrition Survey Report: Lowland and Mountainous Ecological Zones Hajja Governorate Yemen*.

[15] Katuli S., Natto Z. S., Beeson L., Cordero-MacIntyre Z. R. (2013). Nutritional status of highland and lowland children in Ecuador. *Journal of Tropical Pediatrics*.

[16] Ishengoma R. C., Katani J. Z., Abdallah J. M., Haule O., Deogratias K., Olomi J. S. (2016). *Kilosa District Harvesting Plan*.

[17] Fisher A. A., Liang J. E., Townsend J., Stoeckel J. E. (1991). *Handbook for Family Operations Research and Design*.

[18] WHO (1995). Physical status: the use and interpretation of anthropometry. Report of a WHO expert committee. *WHO Technical Report*.

[19] WHO (2006). *WHO Child Growth Standards: Length/Height-for-Age, Weight-for-Age, Weight-for-Length, Weight-for-Height and Body Mass Index-for-Age: Methods and Development*.

[20] Roba K. T., O’Connor T. P., Belachew T., O’Brien N. M. (2016). Variations between post-and pre-harvest seasons in stunting, wasting, and infant and young child feeding (IYCF) practices among children 6–23 months of age in lowland and midland agro-ecological zones of rural Ethiopia. *The Pan African Medical Journal*.

[21] Mgongo M., Chotta N. A. S., Hashim T. H. (2017). Underweight, stunting and wasting among children in Kilimanjaro region, Tanzania; a population-based cross-sectional study. *International Journal of Environmental Research and Public Health*.

[22] WHO (2010). *Nutrition Landscape Information System (NLIS) Country Profile Indicators: Interpretation Guide*.

[23] Kulwa K. B. M., Mamiro P. S., Kimanya M. E., Mziray R., Kolsteren P. W. (2015). Feeding practices and nutrient content of complementary meals in rural central Tanzania: implications for dietary adequacy and nutritional status. *BMC Pediatrics*.

[24] Prendergast A. J. (2015). Malnutrition and vaccination in developing countries. *Philosophical Transcations of the Royal Society B Biological Sciences*.

[25] Ndakidemi P. A., Semoka J. M. R. (2006). Soil fertility survey in Western Usambara mountains, Northern Tanzania. *Pedosphere*.

[26] World Health Organization (2008). *Indicators for Assessing Infant and Young Child Feeding Practices*.

[27] WHO (2009). *Infant and Young Child Feeding: Model Chapter for Textbooks for Medical Students and Allied Health Professionals*.

[28] Tanzania Food and Nutrition (2013). *Infant and Young Child Feeding*.

[29] Christian P., Lee S. E., Donahue Angel M. (2013). Risk of childhood undernutrition related to small-for-gestational age and preterm birth in low- and middle-income countries. *International Journal of Epidemiology*.

[30] Arifeen S. E., Black R. E., Caulfield L. E. (2000). Infant growth patterns in the slums of Dhaka in relation to birth weight, intrauterine growth retardation, and prematurity. *The American Journal of Clinical Nutrition*.

[31] Black R. E., Victora C. G., Walker S. P. (2013). Maternal and child undernutrition and overweight in low-income and middle-income countries. *The Lancet*.

[32] Mborea L. E. G., Bwana V. M., Rumisha S. F. (2015). Malaria, anaemia and nutritional status among schoolchildren in relation to ecosystems, livelihoods and health systems in Kilosa district in central Tanzania. *BMC Public Health*.

[33] Sunguya B. F., Zhu S., Mpembeni R., Huang J. (2019). Trends in prevalence and determinants of stunting in Tanzania: an analysis of Tanzania demographic health surveys (1991–2016). *Nutrition Journal*.

[34] Wemakor A., Garti H., Azongo T., Garti H., Atosona A. (2018). Young maternal age is a risk factor for child undernutrition in Tamale metropolis, Ghana. *BMC Research Notes*.

[35] Romero C. X., Duke J. K., Dabelea D., Romero T. E., Ogden L. G. (2012). Does the epidemiologic paradox hold in the presence of risk factors for low birth weight infants among Mexican-born women in Colorado?. *Journal of Health Care for the Poor and Underserved*.

[36] Khanal V., Sauer K., Karkee R., Zhao Y. (2014). Factors associated with small size at birth in Nepal: further analysis of Nepal demographic and health survey 2011. *BMC Pregnancy Childbirth*.

[37] Rahman M. S., Howlader T., Masud M. S., Rahman M. L. (2016). Association of low-birth weight with malnutrition in children under five years in Bangladesh: do mother’s education, socio-economic status, and birth interval matter?. *PLoS One*.

[38] Patel K. A., Langare S. D., Naik J. D., Rajderkar S. S. (2013). Gender inequality and bio-social factors in nutritional status among under five children attending anganwadis in an urban slum of a town in Western Maharashtra, India. *Journal of Research in Medical Sciences the Offical Journal Isfahan University of Medical Sciences*.

[39] Babatunde R. O., Olagunju F. I., Fakayode S. B., Sola-Ojo F. E. (2011). Prevalence and determinants of malnutrition among under-five children of farming households in Kwara state, Nigeria. *Journal of Agricultural Science*.

